# Molecular docking as a tool for the discovery of novel insight about the role of acid sphingomyelinase inhibitors in SARS- CoV-2 infectivity

**DOI:** 10.1186/s12889-024-17747-z

**Published:** 2024-02-06

**Authors:** Samar Sami Alkafaas, Abanoub Mosaad Abdallah, Mai H. Hassan, Aya Misbah Hussien, Sara Samy Elkafas, Samah A. Loutfy, Abanoub Mikhail, Omnia G. Murad, Mohamed I. Elsalahaty, Mohamed Hessien, Rami M. Elshazli, Fatimah A. Alsaeed, Ahmed Ezzat Ahmed, Hani K. Kamal, Wael Hafez, Mohamed T. El-Saadony, Khaled A. El-Tarabily, Soumya Ghosh

**Affiliations:** 1 Molecular Cell Biology Unit, Division of Biochemistry, Department of Chemistry, Faculty of Science, Tanta University, Tanta, 31527 Egypt; 2 Narcotic Research Department, National Center for Social and Criminological Research (NCSCR), Giza, 11561 Egypt; 3 Biotechnology department at Institute of Graduate Studies and Research, Alexandria University, Alexandria, Egypt; 4 Production Engineering and Mechanical Design Department, Faculty of Engineering, Menofia University, Menofia, Egypt; 5 Faculty of Control System and Robotics, ITMO University, Saint-Petersburg, 197101 Russia; 6 Virology and Immunology Unit, Cancer Biology Department, National Cancer Institute, Cairo University, Cairo, Egypt; 7 Nanotechnology Research Center, British University, Cairo, Egypt; 8 Department of Physics, Faculty of Science, Minia University, Minia, Egypt; 9 Division of Biochemistry, Department of Chemistry, Faculty of Science, Tanta University, Tanta, 31527 Egypt; 10 Biochemistry and Molecular Genetics Unit, Department of Basic Sciences, Faculty of Physical Therapy, Horus University - Egypt, New Damietta, 34517 Egypt; 11 Department of Biology, College of Science, King Khalid University, Muhayl, Saudi Arabia; 12 Biology Department, College of Science, King Khalid University, Abha, 61413 Saudi Arabia; 13 Anatomy and Histology, Faculty of Pharmacy, King Abdulaziz University, Jeddah, 21589 Saudi Arabia; 14 NMC Royal Hospital, 16Th Street, 35233 Khalifa City, Abu Dhabi, United Arab Emirates; 15 Medical Research Division, Department of Internal Medicine, The National Research Centre, 12622 33 El Buhouth St, Ad Doqi, Dokki, Cairo Governorate, Egypt; 16 Department of Agricultural Microbiology, Faculty of Agriculture, Zagazig University, Zagazig, 44511 Egypt; 17 Department of Biology, College of Science, United Arab Emirates University, Al-Ain, 15551 United Arab Emirates; 18 Department of Genetics, Faculty of Natural and Agricultural Sciences, University of the Free State, Bloemfontein, 9301 South Africa; 19 Natural & Medical Science Research Center, University of Nizwa, Nizwa, Oman; 20 Faculty of Physics, ITMO University, Saint Petersburg, Russia

**Keywords:** Ceramide, Sphingomyelin, FIASMAs, ASMase, COVID-19

## Abstract

Recently, COVID-19, caused by severe acute respiratory syndrome coronavirus 2 (SARS-CoV-2) and its variants, caused > 6 million deaths. Symptoms included respiratory strain and complications, leading to severe pneumonia. SARS-CoV-2 attaches to the ACE-2 receptor of the host cell membrane to enter. Targeting the SARS-CoV-2 entry may effectively inhibit infection. Acid sphingomyelinase (ASMase) is a lysosomal protein that catalyzes the conversion of sphingolipid (sphingomyelin) to ceramide. Ceramide molecules aggregate/assemble on the plasma membrane to form “platforms” that facilitate the viral intake into the cell. Impairing the ASMase activity will eventually disrupt viral entry into the cell. In this review, we identified the metabolism of sphingolipids, sphingolipids' role in cell signal transduction cascades, and viral infection mechanisms. Also, we outlined ASMase structure and underlying mechanisms inhibiting viral entry 40 with the aid of inhibitors of acid sphingomyelinase (FIASMAs). In silico molecular docking analyses of FIASMAs with inhibitors revealed that dilazep (S = − 12.58 kcal/mol), emetine (S = − 11.65 kcal/mol), pimozide (S = − 11.29 kcal/mol), carvedilol (S = − 11.28 kcal/mol), mebeverine (S = − 11.14 kcal/mol), cepharanthine (S = − 11.06 kcal/mol), hydroxyzin (S = − 10.96 kcal/mol), astemizole (S = − 10.81 kcal/mol), sertindole (S = − 10.55 kcal/mol), and bepridil (S = − 10.47 kcal/mol) have higher inhibition activity than the candidate drug amiodarone (S = − 10.43 kcal/mol), making them better options for inhibition.

## Introduction

Recently, the world and public health organizations directed resources to curb the outbreak of coronavirus disease (COVID-19) caused by SARS-CoV-2 and its mutated strains [1–6]. Symptoms of COVID-19 infection included respiratory system complications and severe pneumonia, where patients needed intensive medical care and ventilator treatment [5, 7, 8]. The death rate from COVID-19 is about 0.66%, which rises sharply to 7.8% in patients over 80 years old [9]. Severe cases are characterized by a high incidence of cytokine storms and excessive inflammation with high levels of interleukin (IL)-6, IL-8, IL-10, IL-2R, and tumor necrosis factor (TNF)-alpha. The SARS-CoV-2 infects cells by attachment to its particular cellular receptor ACE-2 via a surface unit (S1) of the viral spike glycoprotein [8, 10]. Transmembrane serine protease 2 (TMPRSS2) or cathepsin L cleaves the viral spike protein after entry. When a virus enters host cells, SARS-CoV-2 RNA is released, translation of viral RNA genome into polyproteins is followed by viral release, and then replicate-transcriptase complex is brought together following protein cleavage to promote viral transcription and replication [11].

Previous membrane and cellular changes facilitating SARS-CoV-2 entry may be a promising target to minimize and inhibit viral infection. Lysosomal acid sphingomyelinase is one of the significant signalling molecules in the outer cell membrane and lysosomes [12]. This review focused on sphingomyelinase (ASMase), which converts the sphingolipid (sphingomyelin) into ceramide, which substantially affects the biophysical characteristics of the plasma membrane [13].

Acid sphingomyelinase and ceramide are essential in receptor signalling and infection biology. The acid sphingomyelinase is a glycoprotein lysosomal hydrolase enzyme that catalyzes the degradation of sphingomyelin to phosphorylcholine and ceramide. Although acid sphingomyelinase is found in lysosomes, it is recycled to the plasma membrane because these compartments constantly recycle to the plasma membrane. The activity of acid sphingomyelinase induces ceramide formation in the outer leaflet of the cell membrane. Ceramide molecules generation within the outer leaflet alters the biophysical properties of the plasma membrane because the very hydrophobic ceramide molecules spontaneously associate with each other to form small ceramide-enriched membrane domains that fuse and form large, highly hydrophobic, tightly packed, gel-like ceramide-enriched membrane domains [14].

The conversion of the sphingomyelin in rafts to ceramide can result in raft enlargement, receptor clustering, membrane invagination, and macropinosome formation, all of which promote the uptake of particles, including viruses, into cells and increase viral infectivity. Furthermore, ceramide-enriched membrane domains can bind to proteins and promote viral infectivity. SARS-CoV-2 docks onto ACE2, which is a lipid raft protein. After binding to ACE2, the S protein in the viral envelope undergoes enzymatic activation by TMPRSS2 or furin, likely located in lipid rafts. Subsequent endocytosis of SARS-CoV-2 occurs using a raft-dependent endocytic pathway.

SARS-CoV-2 induces the activity of ASMase and releases the ceramide content in lipid rafts, resulting in the virus's attachment to its receptors and increasing the concentration of virus attachment in lipid rafts domains and viral infectivity. Several reports show that the ASMase/ceramide system controls viral infection. Viruses including Rhinovirus, Ebola, and measles encephalitis [15–17], and bacteria like *Pseudomonas aeruginosa*, *Staphylococcus aureus*, *Salmonella typhi*, and *Neisseria gonorrhoeae* [18–23], stimulate the viral ASMase/ceramide system inducing the development of platform domains rich in ceramide, which facilitate viral entry and host cell infection. As with other viruses, SARS-CoV-2 activates the ASMase/ceramide system, inducing ceramide-enriched-platform formation and facilitating viral entry by clustering ACE-2, resulting in host cell infection [24]. Since 1970, research has shown that weak bases constrain ASMase activity [25]. Weak bases are protonated and diffused into lysosomes, where they are trapped, accumulating intra-lysosomal weakly basic molecules [26]. 

FIASMA are weak bases and accumulate in acidic compartments like the lysosome because they become protonated at the acidic pH. Due to the positive charge, they can no longer cross the membrane (acidic trapping). Consequently, lysosomal ASMase is displaced from the inner lysosomal membrane, and ASMase is proteolyzed. The ASMase/ceramide system is considered a treatment option in patients with respiratory COVID-19 or mutated strains [27]. This review demonstrates the metabolism and importance of sphingolipids responsible for viral infection. The function of ASMase in viral entry and infection is clarified. Accordingly, this review categorizes types of ASMase inhibitors, the functional inhibitors of acid sphingomyelinase (FIASMA) that potentially block viral entry. Additionally, molecular docking in silico of ASMase/ceramide system inhibitors is performed to predict the prospective efficacy of inhibitors as anti-SARS-CoV-2 medication.

## Structure of Sphingomyelinase

**Fig. 1:**
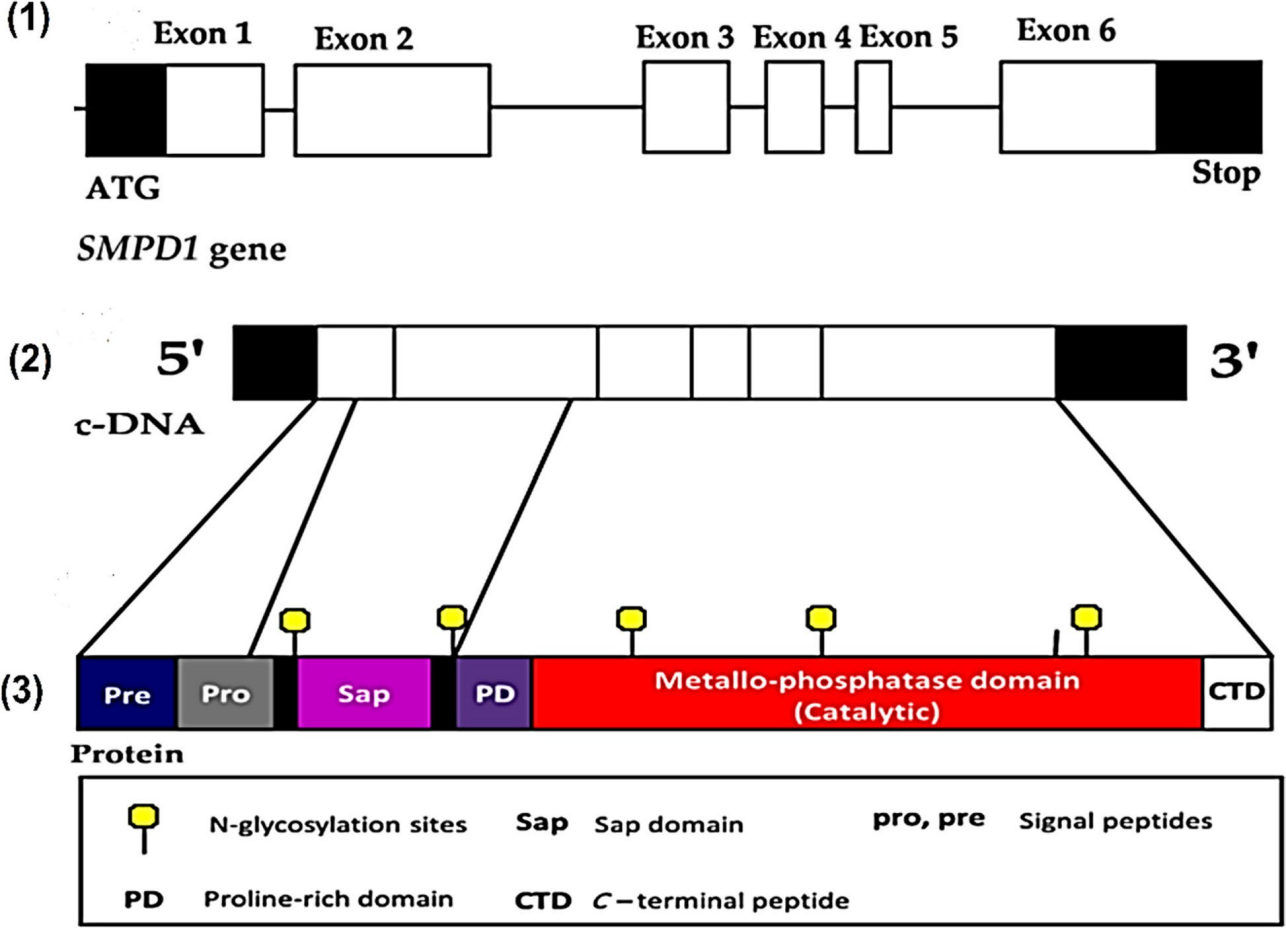
Acid Human Sphingomyelinase (ASMase) exons (1), cDNA (2), and protein structure (3)

Human acid sphingomyelinase is a cellular phosphodiesterase or phospholipase C (PLC), which causes sphingomyelin to hydrolyze into ceramide and phosphocholine by cleavage of the phosphodiester bond. The *SMPD1* gene encodes human ASMase in the chromosomal region 11p15.4 with 6 exons, as shown in Fig. 1 (1). The 1890 bp open reading frame of the whole cDNA for ASMASE codes for 629 amino acids. A monomeric glycoprotein with a protein core of 64 kDa makes up the mature ASMase enzyme. The ASMase enzyme contains 8 disulfide bridges, 5 N-glycosylation sites are occupied, and one N-glycosylation site is not occupied [28, 29], as shown in Figs. 1 (2) and (3).

According to the UniProt blast site, mature ASMase has numerous active domains, including a signal peptide (amino acids 1–46), a Sap-domain (amino acids 89–165), a proline-rich linker domain (amino acids 166–198), the catalytic metallo-phosphatase domain (amino acids 199–461), and the C-terminal domain (amino acids 462–629) [30]. Even in the absence of exogenous sphingolipid activator proteins, the basic sphingomyelinase cleaving activity of the ASMase polypeptide is maintained by its N-terminal Sap-domain [31]. Sphingomyelin attaches to the active site of the catalytic metallo-phosphatase domain, which has a binuclear zinc core, to activate the hydrolysis process and cleave the phosphodiester bond. The ASMase activity depends on the Sap-domain [30, 32, 33].

Human sphingomyelinase is produced in the endoplasmic reticulum as a pre-pro-enzyme with a core protein of 75 kDa, which is quickly cleaved into 72 kDa pro-ASMase in the endoplasmic reticulum-Golgi complex. After cleavage, the pro-ASM is transmitted by the secretory pathway to the extracellular space or endolysosomal compartments. ASMase and numerous other lysosomal hydrolases are transported from the trans-Golgi network (TGN) to late endosomes and lysosomes by the mannose-6-phosphate receptor (M6PR). The ASM lipid-binding proteins, prosaposin, and GM2AP have an alternative route depending on sortilin [34–36].

## Sphingolipid Metabolism

**Fig. 2:**
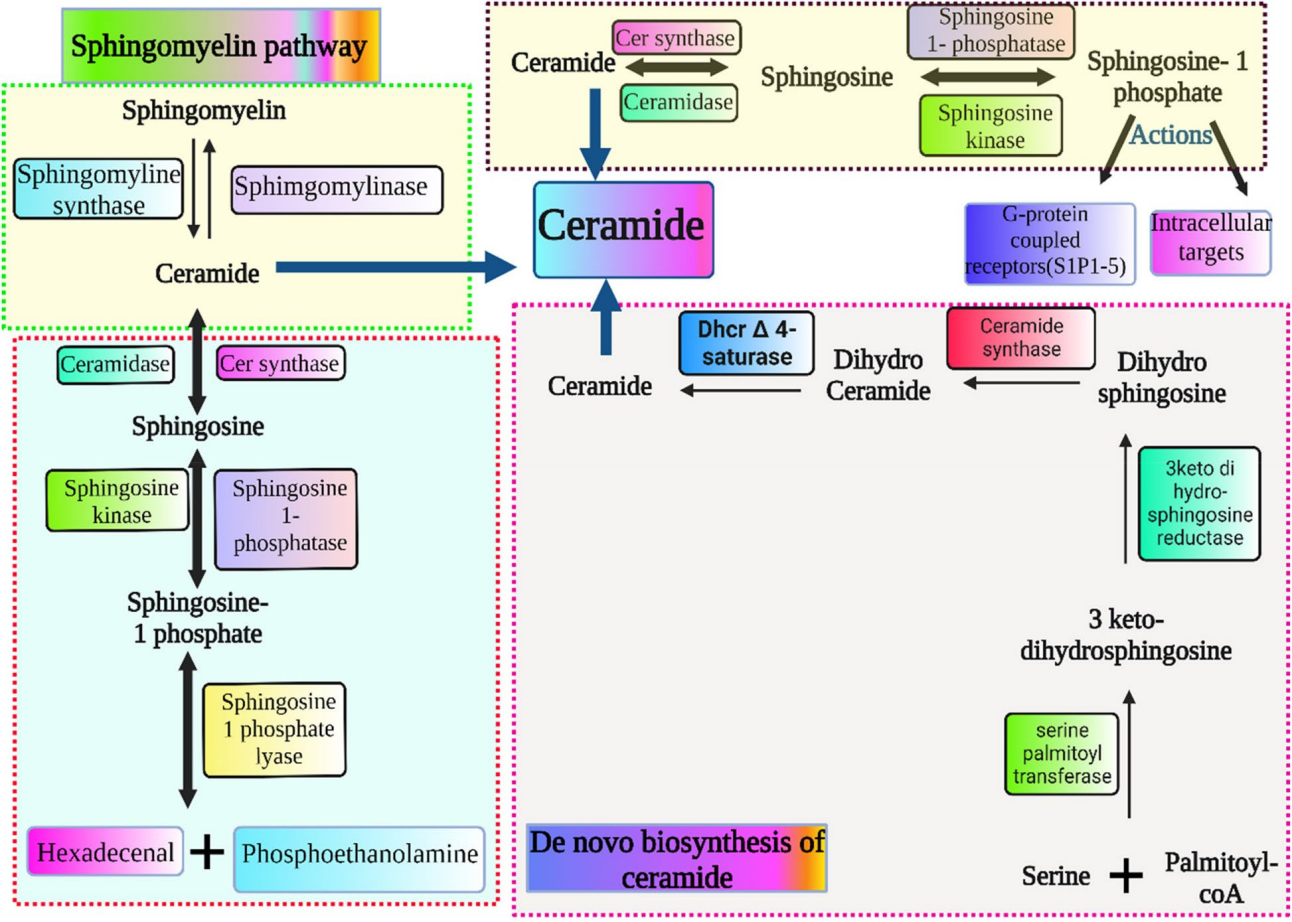
Illustration of sphingolipid metabolism pathway including denovo synthesis and breakdown

Sphingoid bases are the basic structure of sphingolipids, including sphingosine, an 18-carbon unsaturated amino alcohol, the most common among mammals, amid links fatty acids to sphingosine, resulting in ceramide [37]. Sphingomyelin is produced when ceramide is phosphocholine esterified, while glycosylceramides are produced when ceramide is glycolyzed. Sialic acid residues result in ganglioside synthesis, as shown in Fig. 2. These are important cell membrane molecules, and the pathway intermediates for sphingolipid production and breakdown modify processes like apoptosis and T-cell trafficking [37, 38].

Sphingosine, phytosphingosine, and digydrosphingosine represent the first step in creating complex molecules. Sphingosine 1-phosphate, phytosphingosine 1-phosphate, and dihydrosphingosine-1-phosphate are three crucial signalling molecules broken down by phosphorylation of the C1 hydroxyl group. The glycosphingolipids contain a wide range of sphingolipids that differ by the type and arrangement of sugar residues linked to their head groups.

The sphingolipid metabolic pathway is a vital cellular process where ceramide plays an important role in other molecules' metabolism, catabolism, and biosynthesis. Through de novo synthesis, sphingolipids are produced via serine and palmitoyl CoA condensation. This process is catalyzed by serine palmitoyl transferase, which results in 3-keto-dihydrosphingosine [39]. Hydrolysis of sphingomyelin by sphingomyelinase into ceramide keeps the membranes in homeostasis conditions. Thus, sphingolipid metabolism is complicated but involves the de novo biosynthesis of ceramide in the endoplasmic reticulum. Ceramide is the key product in the breakdown of sphingomyelin or their de novo synthesis, which is the process by which sphingolipids are metabolically processed. The de novo synthesis could begin with serine palmitoyl-transferase, serine condensation, and palmitoyl–coenzyme A to 3-keto di hydrosphingosine [37]. Then, the reduction of 3-ketodihydrosphingosine into sphinganine is carried out by 3-ketodihydrosphingosine reductase.

Ceramide synthase adds acyl fatty acids to sphinganine, leading to dihydroceramide production. In the endoplasmic reticulum, dihydroceramide D4 saturates and desaturates into ceramide. A ceramide transfer protein transports ceramide from the endoplasmic reticulum to the Golgi apparatus. Sphingosine (2 amino-4-trans-octadecene-1,3-diol) is produced from ceramide by ceramidase enzymes. Ceramide synthase is responsible for the production of ceramide in a way opposite to ceramidase enzymes. Sphingosine kinase 1 (SPhK1) or sphingosine kinase 2 (SPhK 2) phosphorylate sphingosine to produce sphingosine 1-phosphate (S1P). Sphingosine is phosphorylated into sphingosine 1-phosphate (S1P) by either sphingosine kinase 1 (SPhK1) or sphingosine kinase 2 (SPhK 2). The S1P phosphatases transform S1P back to sphingosine, or the S1P lyase enzyme breaks down S1P into hexadecanal and phosphoryl ethanolamine [37]. Sphingolipids are generated from ceramides by sphingomyelin synthase, while sphingomyelin is converted to ceramide via sphingomyelinase. The sphingolipid metabolism pathway is illustrated in Fig. 2.

## Sphingolipid Transport and Uptake

Bioactive sphingolipids interact with mediators to produce functional responses. Sphingolipids include sphingomyelin, ceramides, sphingosine, and S1P [40]. Sphingosine contains one aliphatic chain that usually has 18 carbon atoms along its length, enabling easy passage between distinct membranes. Sphingosine 1 phosphate is generated in the inner cell plasma membrane in response to tumor necrosis factor-cytokine as signalling (TNFa). Then, it transfers to the outer leaflet of the plasma membrane to bind to its S1PRs receptor [41]. The ABC transporter superfamily has two members proposed to control S1P trafficking [42]. There are two members to regulate S1P, either internalization or efflux by cystic fibrosis transmembrane regulator (CFTR) or ABCC1.

Sphingomyelin contains two aliphatic chains and a zwitter ionic head group. Thus, it has little aqueous solubility and hardly ever flip-flops across bilayers but moves laterally [43]. The movement of sphingomyelin may be hampered by interactions with sterols in cell membranes and self-aggregation [43]. The ceramide structure has two aliphatic chains and a neutral head group. Ceramide is transported from its production site in the endoplasmic reticulum to the Golgi apparatus under the control of the Ceramide Transfer Protein (CERT). Studies show neutral ceramide easily flip-flopping across cell membranes [43].

It is unknown if the organization of ceramide into microdomains prevents ceramide from flipping from the outer leaflet to the inner leaflet of the plasma membrane or whether ceramide can flip-flop as effectively in complex biological membranes [43]. Limiting the flipping of ceramide could impact its signalling functions significantly.

## Role of Sphingolipids in Viral Entry

Lipid rafts are particular regions of the host cell membrane that are profuse in lipids like cholesterol, sphingolipids, and gangliosides [44]. These lipid-rich domains are characterized by containing well-organized lipid molecules stacked tightly. Studies show lipid rafts are key in viral infection cycles, including HIV, poliovirus, hepatitis C, and coronaviruses [45–47]. The SARS-CoV-2 virus uses lipid rafts and caveolae-mediated endocytosis for viral entry [48]. Thorp and Gallagher (2004) observed that methyl-β-cyclodextrin triggers cholesterol depletion and inhibits viral entry and infection. This observation supports a lipid raft’s functional role in viral entry [49]. Coronaviridae, including SARS-CoV, use lipid rafts to enter and host infection. In addition to the minor envelope protein and membrane protein, the SARS-CoV-2 envelope contains spike protein (S) [50]. A viral spike (S) comprises S protein trimmers, which act in viral fusion with host cellular membranes and constitute two subunits (S1 and S2). After viral binding, spike protein is cleaved by host protease transmembrane serine protease 2 with furin pre-cleavage to facilitate viral entry [11, 51, 52]. SARS-CoV-2 entry is receptor-mediated endocytosis through a specific host receptor (ACE-2). Viral S protein binds with ACE-2, enabling proteolysis of viral S1 protein by host proteases, which may be attached to caveolae, including TMPRSS2 and Cathepsin L [53].

Moreover, SARS-CoV-2's ability to enter and cause host infection depends on its interaction with specific gln493 residue of the ACE-2 receptor [54]. Viral entrance may be mediated by the host ACE-2 receptor or by sialic acids interacting with host cell surface ganglioside binding domains. This domain (111–158) is a well-conserved sequence causing viral attachment to lipid rafts, which makes it easier for SARS-CoV-2 to infect the host's ACE-2 receptor [54]. ACE-2 must colocalize with the raft markers GM1 and caveolin-1. Lipid rafts are a key platform that can concentrate host ACE-2 receptors interacting with viral S protein. Viral particles can bind to the surface of the host cell membrane because ACE-2 clusters in certain positions in the cell membrane. In this approach, lipid raft microdomains boost the efficacy of viral infection. These results agree with cholesterol depletion and reduce, but do not prevent, the susceptibility to viral infection [55].

Lipid rafts are considered targets for inhibiting viral infection. Drugs such as methyl-β-cyclodextrin cause disruption of lipid rafts, resulting in viral entry inhibition [56]. Pathogen-host interactions probably aid the development of focal adhesions and lipid raft clustering during endocytosis. Table 1 shows other inhibitors of lipid rafts such as propofol, isoflurane, pentobarbital, aspirin, naproxen, perifosine cisplatin, azithromycin, daunorubicin, doxorubicin, quercetin, and luteolin. These inhibitors may be used as antiviral drugs against SARS-CoV-2. Thus, research on lipid rafts should be included in developing antiviral drugs.

**Table 1:** Common inhibitors of lipid rafts with their mechanism

Drug	Mechanism	References
Propofol	The propofol has a role for caveolae (specifically caveolin-1) in propofol-induced bronchodilatation. Due to its lipid nature, propofol may transiently disrupt caveolar regulation, thus altering ASM [Ca^2+^] and decreasing caveolin-1 expression	[57]
Isoflurane	The isoflurane increases membrane fluidity and the permeability of the blood–brain barrier by distributing the highly ordered lipid domains with saturated lipids. It also weakened the sterol-phospholipid association in cholesterol-rich membranes	[58, 59]
Pentobarbital	Pentobarbitals modify the physical characteristics of lipid rafts on model membranes and cause lipid membrane disorder of brain plasma membranes	[60]
Lidocaine	Lidocaine is observed to distribute the erythrocyte membrane lipid rafts reversibly and abolish flotillin-1 in lipid rafts together with depleting cholesterol. In addition, the Lidocaine hydrochloride, an amphipathic local anaesthetic, is shown to reversibly disrupt rafts in erythrocyte membranes and alter the Gsα dependent signal transduction pathway. These findings provide evidence of rafts' presence while maintaining normal cholesterol content in erythrocyte membranes and confirm a role for raft-associated Gsα in signal transduction in erythrocytes	[61, 62]
Tetracaine	Tetracaine induces lipid chain mobility, destabilizes the supported lipid bilayers, and induces lipid raft distribution and solubilization. Tetracaine causes a curvature change in the bilayer, which leads to the formation of the subsequent formation of up to 20-μm-long flexible lipid tubules as well as the formation of micron-size holes	[63]
Dibucaine	Dibucaine hydrochloride has a distribution effect on lipid rafts. The inserting Dibucaine molecules into lipid bilayers induces a reduction in the ternary liposome's miscibility transition temperature (Tc) and a reduction in the phase boundary line tension. This suggests that the Dibucaine.HCl molecules may disturb ion channel functions by affecting the lipid bilayers surrounding the ion channels	[64]
Bupivacaine	Bupivacaine stereostructure specifically interacts with membranes containing cholesterol, which is consistent with the clinical features of S (-)-bupivacaine. The bupivacaine interacted with liposomal membranes to increase membrane fluidity. They also revealed that the interactivity with lipid bilayer membranes is largely consistent with the local anaesthetic potency	[65]
Dexmedetomidine Levomedetomidine Clonidine	Dexmedetomidine and clonidine acted on lipid bilayers to increase the membrane fluidity with potencies varying by a compositional difference of membrane lipids. Dexmedetomidine showed greater interactivity with neuro-mimetic and cardiomyocyte-mimetic membranes than clonidine, consistent with their comparative lipophilicity and activity. The effects of α_2_-adrenergic agonists on lipid raft model membranes were much weaker than those on other membranes, indicating that lipid rafts are not mechanistically relevant to them. Higher interactive dexmedetomidine was discriminated from lower interactive levomedetomidine in the presence of chiral cholesterol in membranes. An interactivity difference between the two enantiomers was largest in the superficial region of lipid bilayers, and the rank order of their membrane-interacting potency was reversed by replacing cholesterol with epicholesterol, suggesting that cholesterol’s 3β-hydroxyl groups positioned close to the membrane surface are responsible for the enantioselective interaction	[66]
Morphine	Morphine increases the membrane fluidity of membranes	[67]
Aspirin	It is observed that aspirin increases membrane fluidity, disrupts the membrane organization, and prevents raft formation	[64]
Indomethacin Naproxen Ibuprofen	These compounds affected the organization of rat-like ordered lipid and protein membrane nanoclusters	[68]
Edelfosine	It is observed that Edelfosine increases the fluidity of lipid rafts. Edelfosine is associated with cholesterol and colocalizes in vivo with rafts, causing the raft's structure modification	[69]
Perifosine	It is observed that perifosine causes disrupted membrane raft domains	[70]
Edelfosine Miltefosine	The edelfosine and miltefosine increase the fluidity of raft model membranes	[71]
Erucylphosphocholine	Erucylphosphocholine is observed to increase the membrane raft fluidity and weaken the interaction between cholesterol and sphingomyelin	[72]
2-Hydroxyoleic acid	2-Hydroxyoleic acid increases the membrane raft fluidity	[73]
Cisplatin	Cisplatin increases the membrane fluidity and induces apoptosis, which was inhibited by cholesterol (30 μg/mL) and monosialoganglioside-1 (80 μM)	[74, 75]
Azithromycin	Azithromycin is observed to increase the fluidity of raft-like membranes	[76]
Daunorubicin	Daunorubicin is observed to affect lipid rafts by decreasing the fluidity of raft-like membranes	[77]
Doxorubicin	Doxorubicin is an anticancer drug that increases the fluidity of binary membranes but not ternary membranes	[78]
Quercetin	Quercetin is observed to suppress the accumulation of lipid rafts to inhibit TNF-α production. In addition, it increases the fluidity of raft model membranes in mouse macrophages	[79, 80]
Luteolin	Luteolin suppresses the accumulation of lipid rafts to inhibit TNF-α production in mouse macrophages	[80]
EGCG	Epigallocatechin gallate (EGGG) decreases the fluidity of binary membranes. On the other hand, it induces lipid raft clustering and apoptotic cell death in human multiple myeloma cells	[81]
Dimeric procyanidin	Dimeric procyanidin increases the membrane fluidity in human acute T-cell leukemia cells	[82]
Hexameric procyanidin	Hexameric procyanidin decreases the membrane fluidity and prevents the lipid raft disruption induced by deoxycholate in human colon cancer cells	[83]
Emodin	Emodin causes disrupted lipid rafts in human umbilical vein endothelial cells	[84]
Ginsenosides	Ginsenosides increase the membrane fluidity and reduce the raft-marker protein concentration in lipid rafts in HeLa cells	[85]
Saikosaponin	Saikosaponin inhibits Lipopolysaccharide-induced cytokine expression and Toll-like receptor localization in lipid rafts, and reduces membrane cholesterol levels in mouse macrophages	[86]
Methyl-beta-cyclodextrin (MβCD) treatment	It is observed that MβCD causes depletion of cholesterol in the rafts by methyl-beta-cyclodextrin (MβCD) treatment impaired the expression of the cell surface receptor angiotensin-converting enzyme 2 (ACE2), resulting in a significant increase in SARS-CoV-2 entry into cells	[87]
Statins	Statins reduces cholesterol synthesis by inhibiting the activity of HMG-CoA reductase. Statins could modulate virus entry, acting on the SARS‐CoV‐2 receptors, ACE2 and CD147, and/or lipid rafts engagement. In addition, statins, by inducing autophagy activation, could regulate virus replication or degradation, exerting protective effects	[88]

## The Acid Sphingomyelinase/Ceramide System in Viruses

Scientific studies showed severe consequences and harsh symptoms resulting from acute respiratory syndrome coronavirus 2 (SARS-COV-2). Virus infectivity and spread have been extensively studied. Interestingly, SARS-CoV-2 infectivity occurs by attachment to the host cell receptor via S proteins. This results in virus priming by proteases, facilitating viral entry through endocytosis and completing the viral life cycle. The sphingolipid family is the most common lipid along the cell membrane, including sphingosine and ceramide. Such lipids can interfere with virus uptake into epithelial cells and in cultures of human nasal cells. With the different mechanisms of action, sphingosine is blocked, while ceramide enables viral infection. The well-known acid sphingomyelinase (ASMase) is essential to produce ceramide, and drug inhibition, like amitriptyline, reduces entry into epithelial cells.

Consequently, a key prognostic marker for assessing the severity of COVID-19 is S1P [89]. ASMase transforms sphingomyelin into ceramide, found either on the cell membrane surfaces or attaches to the outer surface of plasma membranes. Acid sphingomyelinase surfaces function as signalling molecules and produce ceramide in the outer parts of plasma membranes. The ceramide molecules are hydrophobic and form small membrane domains that rearrange to form larger platforms. These domains recognize 1-integrin, CD95, CD40, DR5, and other activated receptor molecules. Ceramide platforms mediate bacterial or viral infection and other stress stimuli [24].

When viruses enter cell membranes, sphingolipids function as bioactive lipids that transmit signals inside and outside cells. So, limiting viral replication by targeting the host cell's sphingolipid metabolism may give a chance for more therapeutic approaches. Host cell viral infection begins with endocytosis, then un-coating, exocytosis, and discharge of nucleocapsids into the cytoplasm. These previous actions are affected by membrane microdomains. Subsequently, the interactions between viruses and cells promote different signal cascades affecting cellular uptake, intracellular trafficking, and viral replication [90].

The ASMase activity is implicated in other viruses like Ebola’s early infection stages. Acid sphingomyelinase activation is crucial for Ebola virus endocytosis, making Niemann-Pick C protein 1 (NPC1), an endo/lysosomal cholesterol transporter, virus particle-accessible. To facilitate the fusing of the Ebola virus and endosomal membranes, NPC1 is essential for viral absorption. Thus, NPC1 acts as a receptor for the proteolytically activated viral envelope protein in an intracellular compartment rather than at the plasma membrane.

Acid sphingomyelinase activation is also recognized after the interaction of dendritic cells with the measles virus. Viral glycoproteins interact with DC-SIGN on the cell surface, which induces the activation of sphingomyelinase and the release of ceramide molecules. Then, measles virus receptor CD150 entry is translocated from an intracellular storage compartment to the cell surface, favouring viral infection of dendritic cells [90].

## Trafficking Process utilized in viral entry

The trafficking or endocytosis process enables cells to internalize macromolecules, nutrients, or viruses into the cell [91]. The endocytosis process is classified into receptor-mediated endocytosis, caveolae uptake, or clathrin-independent endocytosis, including the CLIC/GEEC pathway [92–94]. Internalized macromolecules are categorized by endosomes, which are a pleiomorphic series of tubulovesicular compartments [95]. Internalized macromolecules are processed in various ways, including back-recycling to the cellular plasma membrane, degradation by delivery to the lysosomal molecules, or to polarized cells through transcytosis [96]. Several events accompany the maturation of endosomal compartments, including luminal pH decrease, significant phosphatidylinositol lipid alterations via regulating lipid kinases and phosphatases, and activation and differential Rab-family GTPase recruitment. The trafficking or endocytosis process has critical cellular functions. Functions include cellular communication between cells and the environment, controlling cellular homeostasis and regulating essential surface proteins, and viral or bacterial entrance [97]. Moreover, the process regulates cell signalling through G-protein coupled receptors and receptor tyrosine kinases [98, 99]. This review focuses on clathrin and dynamin-independent pathways, especially lipid raft entry, and their role in SARS-CoV-2 entry.

## Clathrin and dynamin-independent pathways utilized in viral entry

Receptor-independent endocytosis (CIE) includes the CLIC/GEEC pathway responsible for cellular functions. For instance, cell signalling, adhesion, nutrient receptors, and regulation of the expression of certain membrane transporters. The endocytic vesicles/tubules of CIE are characterized by having no distinct coat. The CIE was discovered using inhibitors blocking clathrin-mediated and caveolae-mediated endocytosis [92, 93, 100]. Small GTPases Rac1 and Cdc42 involved in clathrin- and dynamin-independent pathways are responsible for actin formation-dependent clathrin-independent carriers (CLICs) [101]. The GPI-AP enriched endosomal compartments are specific early endosomal compartments generated by the fusion of CLICs (GEECs) [102, 103]. This process, called the CLIC/GEEC pathway, depends on specific proteins, including GTPase and Arf6, and is responsible for taking and recycling the major Histocompatibility Antigen I [104].

Small protein Arf6 triggers the activation of phosphatidylinositol-4-phosphate 5-kinase, resulting in PI(4,5)P2, which induces actin assembly and drives endocytosis [105]. Another endocytosis pathway is the flotillin pathway, which depends on curvature-generating and membrane-anchored proteins [106, 107]. In vitro HeLa cells undergo CLIC/GEEC and a flotillin-dependent pathway, taking up PI-anchored protein and CD59. The CLIC/GEEC pathway and the Arf6-pathway are both involved in the uptake of the transmembrane protein CD44 [108]. Several types of CLIC/GEEC pathways play a role in rapidly recycling cell membranes. The CLIC/GEEC pathway is responsible for nutrient and toxin uptake and is considered a portal for viral infection [109].

## SARS-CoV-2 Entry by Lipid Rafts

Viral entry and infection depend on endocytosis pathways, especially sphingolipids and lipid rafts. Many viruses utilize lipid rafts to enter the host cell and facilitate infection, including hepatitis C viruses [47], human herpes virus 6 [110], poliovirus [46], and simian virus 40. Coronaviruses, including SARS-CoV-2, interact with lipid rafts to enter host cells and cause infection [111, 112]. Studies by Thorp and Gallagher (2004) supported the function of sphingolipids and cholesterol in viral infections, where cholesterol reduction prevents viral entry [49].

The virus is made up of an envelope that includes spike protein (S), membrane protein (M), and minor envelope protein (E). Transmembrane serine protease 2 (TMPRSS2), with the help of furin, triggers cleavage of the viral spike (S1 and S2) [51]. The Golgi apparatus contains a predominant amount of furin; the other part is found on the cell surface [52]. Once the viral spike and its structural proteins bind to ACE-2, it is activated and promotes viral entry into the host cell.

The host cell receptor is angiotensin-converting enzyme-2 that binds to the S proteins in the virus [113], enabling proteolysis of viral surface S1 subunit by a plasma-membrane-bound serine protease (TMPRSS2) and Cathepsin L (CatL), which may be associated with caveolae [114]. Once SARS-CoV-2 is attached to caveolae and enters intracellular endosomes, cathepsin L emerges as the main protease of the virus [115].

**Fig. 3:**
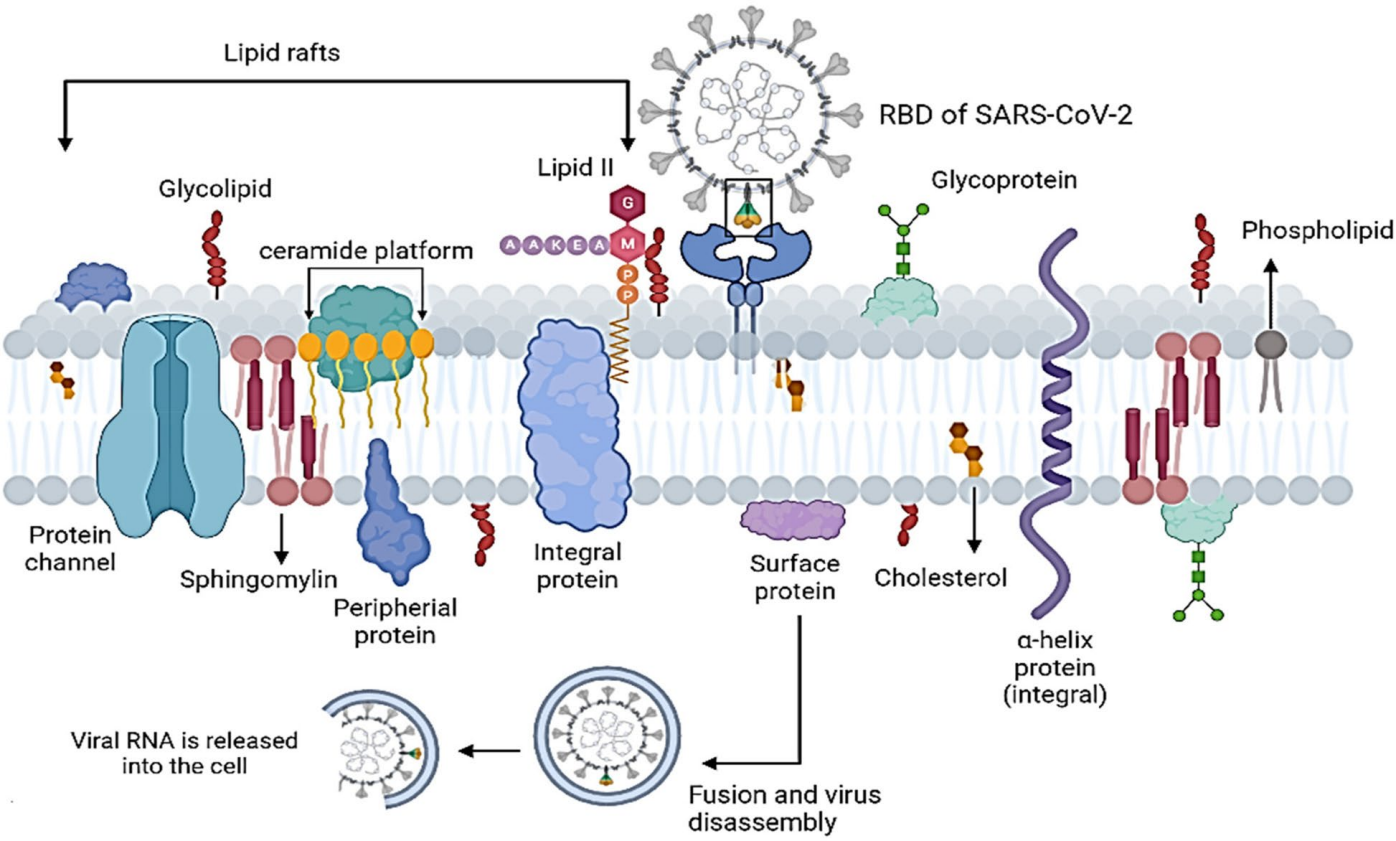
Representation of SARS-CoV-2 Entry mechanism by interacting of spike protein in RBD of the virus with host receptor ACE-2 and consequently internalized into the cell through endocytosis process by helping lipid rafts

Viral gateway into the host cell or ACE-2 receptor exists on the surface of several types of cells, including kidney, respiratory, and intestinal epithelial and endothelial cells. Respiratory SARS-CoV-2 attaches to ACE-2 by gln493 residue, enabling viral entry. Viral S protein not only attaches to ACE-2 but also binds to host cell surface gangliosides. A new type of ganglioside-binding domain (111–158) was identified within the N-terminal domain of the SARS-CoV-2 S protein, facilitating attachment of viral spike to lipid rafts and attachment to host cell receptors [54]. The ACE-2 is colocalized with SARS-CoV-2, entering and infecting host cells by direct membrane fusion or by host cell ACE-2. Lipid rafts are key in both viral entrance methods, enabling the concentration of the endocytic proteins for endocytosis and fusion, as shown in Fig. 3. When endocytic proteins concentrate and interact within lipid rafts, the frequency of interprotein collisions by protein partitioning into lipid rafts increases [116]. As a result, lipid rafts act as plasma membrane "chambers" that facilitate protein interactions on the plasma membrane, promote the rate of molecule collisions, and consequently improve the efficacy of membrane reactions.

Lipid rafts play a role in viral infection by providing appropriate platforms that concentrate host cell receptor ACE-2 on the cell membrane, where they attach with viral S protein. The receptor binding domain (RBD) is the vital part of the virus that engages the protease domain (PD) of ACE-2, resulting in a complex containing a dimeric ACE-2 with two S protein trimers [117]. Multivalent binding of virus particles to the cell surface occurs by host cell receptors clustering. Microdomains of the host cell membrane improve the efficiency of viral infection and facilitate viral endocytosis. Several studies confirm that methyl-β-cyclodextrin (MβCD) inhibits infectious bronchitis virus infection by disrupting lipid rafts, indicating that lipid rafts play a role in viral entry [56, 118]. After the viral S protein attaches to ACE-2 and the virus and host receptor complex have formed, lipid raft and focal adhesions are clustered during endocytosis. Therefore, lipid rafts are hypothesized to be significant during the early stages of coronavirus infection.

## Lipid Raft Distribution Reduces SARS-CoV-2 Infectivity

Some drugs affect lipid rafts and thus play an important role as antiviral drugs. Lipid raft distribution and cholesterol depletion by methyl-β-cyclodextrin (MβCD) minimizes the infectivity of the influenza virus [119]. Lipid raft distribution reduces viral infectivity and holes in the viral envelope, which disturb the viral structure and affect viral protein release. Several studies observed that cholesterol depletion reduces the infectivity of SARS-CoV-2 [120–122]. Inhibiting of viral biosynthesis and infection occurs using drugs such as lovastatin or squalestatin that promote cholesterol depletion. A significant viral ASM/ceramide system in SARS-CoV-2 is important for viral infection. Entry of SARS-CoV-2 and clustering with host cell receptors are facilitated by stimulation of the ASM/ceramide system, subsequently forming membrane domains rich in ceramide platforms on the cell membrane [24].

In this context, the ASM/ceramide system is considered an antiviral target to reduce viral infection. FIASMAs are antiviral drugs used against the ASM/ceramide system in SARS-CoV-2 that inhibit the formation of ceramide-enriched membrane domains, thereby preventing SARS-CoV-2 infection (Table 2). Ceramide has several functions, including clustering of ACE-2 in large membrane domains and amplifying signaling via ACE-2, which is also required for host cell ACE-2 internalization of the virus into the endosome [123]. Cathepsins in the endosome interact with ceramide produced inside endosomes or on the cell membrane's outer leaflet, promoting spike-protein priming and membrane fusion [124]. As a result, FIASMAs inhibit the formation of domains enriched with ceramide and viral entry and infection. In this context, viral infection is inhibited by the down-regulation of the genetic expression of ASMase. FIASMAs change the pH of the endosome, enabling lysosomes to target the endosome and make the virus more susceptible to lysosomal degradation. Therefore, our review suggests FIASMA medications as antiviral therapeutics by targeting lipid raft domains.

**Table 2:** Functional inhibitors of acid sphingomyelinase (FIASMs) with US Food and Drug Administration (FDA) appraisal

No	FIASMAs	FDA	Molecular weight g/mole	References
1	Alverine	Not approved	281.44	[27, 125, 126]
2	Astemizole	Approved	458.571	[127]
3	Aprindine	Not approved	322.487	[27, 125]
4	Amlodipine	Approved	408.879	[125, 127]
5	Ambroxol	Approved	378.1028	[125]
6	Amiodarone	Approved	645.31	[128, 129]
7	Amitriptyline	Approved	277.403	[128–130]
8	Benztropin	Approved	307.429	[127, 131]
9	Bepridil	Approved	366.54	[127, 131, 132]
10	Biperidene	Approved	311.46	[27, 125]
11	Camylofine	Approved	320.47	[127]
12	Carvedilol	Approved	406.474	[27, 125, 128, 130]
13	Cepharanthine	Not approved	606.7	[27, 125, 133, 134]
14	Clofazimine	Approved	473.4	[125, 128]
15	Clemastine	Approved	343.89	[125, 128, 135, 136]
16	Cloperastine	Approved	329.86	[127, 134, 137]
17	Chlorprothixene	Not approved	315.86	[127, 128, 135]
18	Chlorpromazine	Approved	318.86	[128, 131, 134, 138]
19	Clofazimine	Approved	473.39	[128, 139, 140]
20	Clomiphene	Approved	405.966	[127, 141, 142]
21	Clomipramine	Approved	314.9	[128, 131, 143, 144]
22	Conessine	Not approved	356.6	[27, 125]
23	Cyclobenzaprine	Approved	275.4	[127, 128]
24	Cyproheptadine	Approved	287.39	[127, 128]
25	Desipramine	Approved	266.388	[128, 143, 145]
26	Desloratadine	Approved	310.82	[27, 125, 145]
27	Dicycloverine	Approved	309.487	[27, 125, 140]
28	Dilazep	Approved	604.7	[132, 146]
29	Dimebon	Not approved	319.452	[27, 125]
30	Doxepine	Approved	279.376	[127, 132, 147]
31	Drofenine	Approved	317.47	[127, 128, 143, 145]
32	Emetine	Not approved	480.639	[125, 134, 148–150]
33	Fendeline	Approved	315.5	[127]
34	Flupenthixol	Not approved	434.5219	[136, 143, 151]
35	Fluoxetine	Approved	309.33	[127–129, 144, 146]
36	Fluvoxamine	Approved	318.335	[125, 143, 152]
37	Fluphenazine	Approved	437.523	[125, 128, 131, 153]
38	Flupentixol	Not approved	434.5219	[136, 143, 151]
39	Flunarizine	Not approved	404.495	[128, 135]
40	Hydroxyzin	Approved	374.904	[125, 129, 144, 154, 155]
41	Imipramine	Approved	280.407	[128, 143, 145, 156]
42	Loperamide	Approved	477.037	[129, 135, 157]
43	Loratadine	Approved	382.88	[128, 154, 158]
44	Maproteline	Approved	277.403	[127, 128, 135, 145]
45	Melatonine	Not approved	232.278	[130, 138, 159, 160]
46	Mebhydroline	Not approved	276.376	[125]
47	Mebeverine	Not approved	429.55	[27, 125]
48	Mibefradile	Not approved	495.63	[27, 125]
49	Norfluoxetine	Approved	295.30	[127]
50	Nortriptyline	Approved	263.377	[125, 127, 146]
51	Paroxetine	Approved	329.37	[127, 129, 144, 160, 161]
52	Perphenazine	Approved	403.97	[27, 125, 128, 162]
53	Pimozide	Approved	461.56	[27, 125]
54	Pimethexene	Approved	293.434	[127]
55	Profenamine	Discontinued	312.5	[27, 125]
56	Promethazine	Approved	284.4191	[127, 128, 131, 155]
57	Promazine	Not approved	284.42	[127]
58	Protriptyline	Approved	263.377	[127, 128]
59	Quinacrine	Not approved	400.0	[155, 163]
60	Sertindole	Not approved	440.941	[27, 125]
61	Solasodine	Not approved	413.64	[27, 125]
62	Sertraline	Approved	306.229	[127, 144, 164]
63	Suloctidil	Not approved	337.6	[127]
64	Tamoxifene	Approved	371.515	[144, 155, 164]
65	Thioridazine	Approved	370.6	[163–165]
66	Tomatidine	Not approved	415.7	[27, 125]
67	Terfenadine	Not approved	471.673	[127]
68	Trifluoperazine	Approved	407.497	[128, 151, 164]
69	Triflupromazine	Approved	352.4	[125, 128]
70	Trimipramine	Approved	294.434	[128, 166]
71	Zolantidine	Not approved	381.5	[27, 125]

## Functional Inhibitors of Acid Sphingomyelinase FIASMAs’ Mechanism of Action

**Fig. 4:**
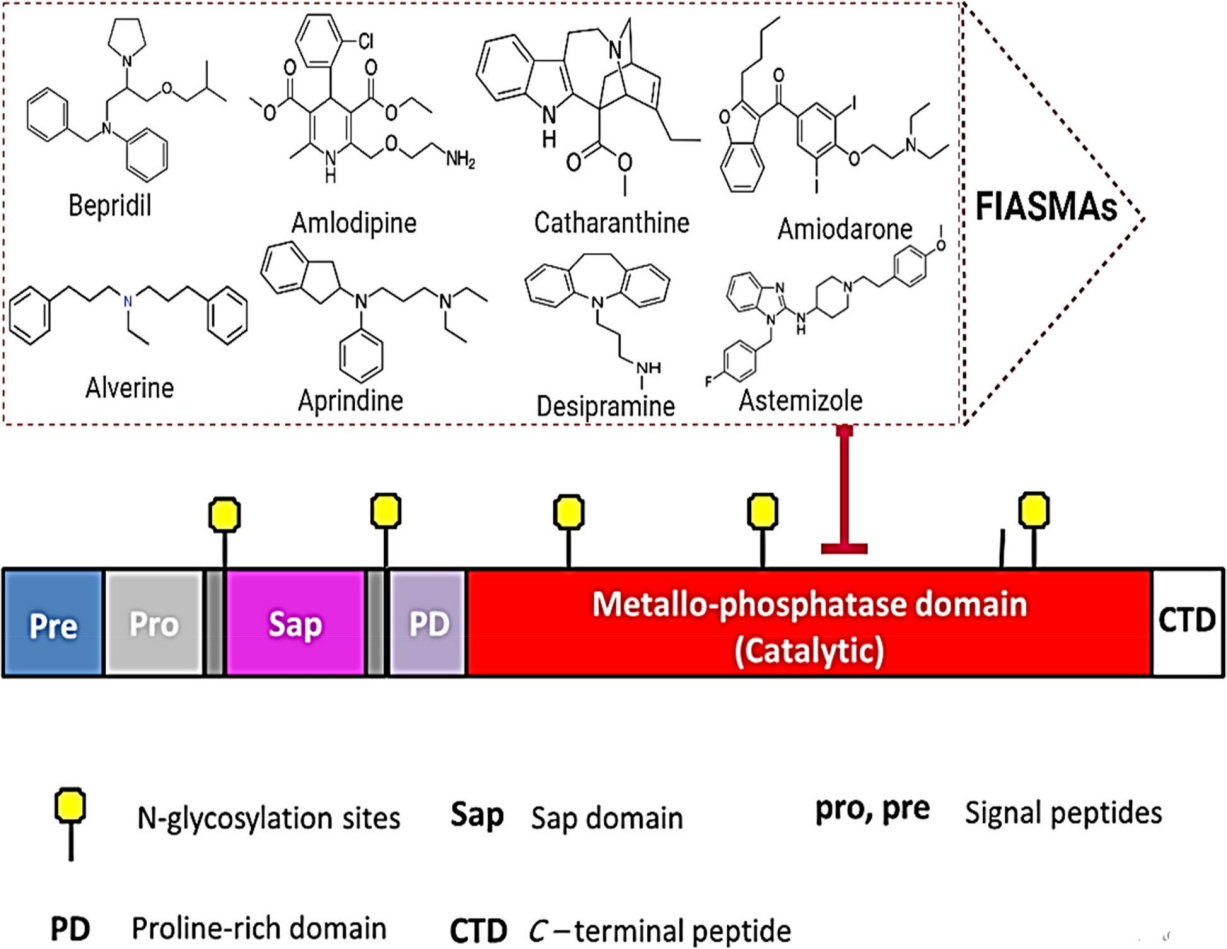
Representation of targeting FIASMAs on the catalytic domain of ASMase protein

Specific electrostatic forces bind lysosomal acid sphingomyelinase to the intra-lysosomal membranes and thus remain protected against proteolytic activity. FIASMAs inhibit ASMase by an indirect mechanism [26] (Figs. 4). The intra-lysosomal space maintains a low pH by an ATP-driven proton pump, which retains the attachment of the ASMase to the intra-lysosomal membranes. The lysosomal membrane is characterized by low permeability towards the protonated bases compared to uncharged ones (lysomotropism). Therefore, with the intake of FIASMAs and other weak bases (lysosomal accumulation), the intra-lysosomal pH raises and diminishes the electrostatic interactions between the lysosomal membrane and the ASMase, resulting in ASMase detachment [127].

**Fig. 5:**
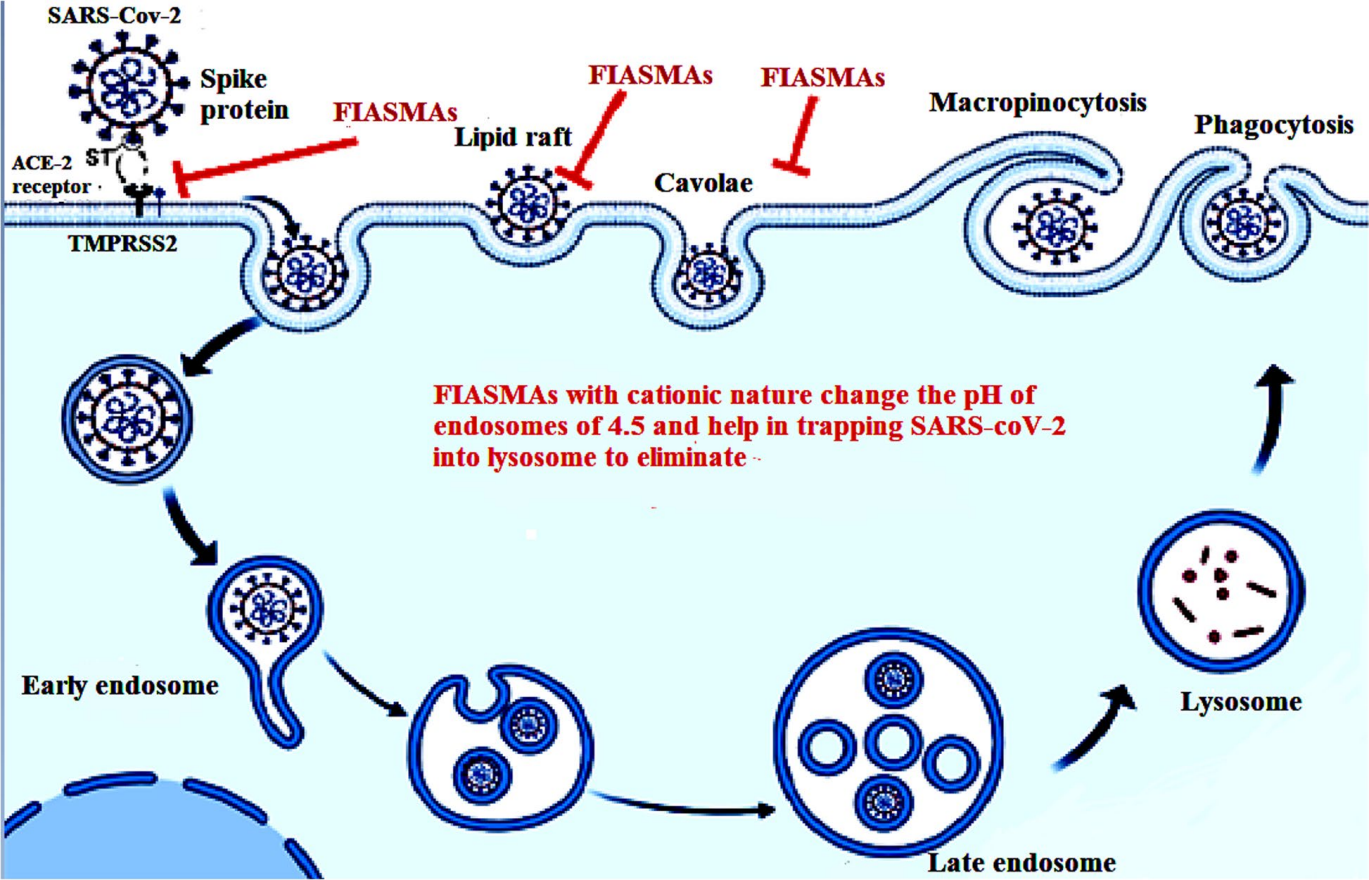
Representation of FIASMAs is antiviral drugs used against ASM/ceramide system in SARS-CoV-2 inhibits the formation of ceramide-enriched membrane domains, thereby preventing infection with SARS-CoV-2

**Fig. 6:**
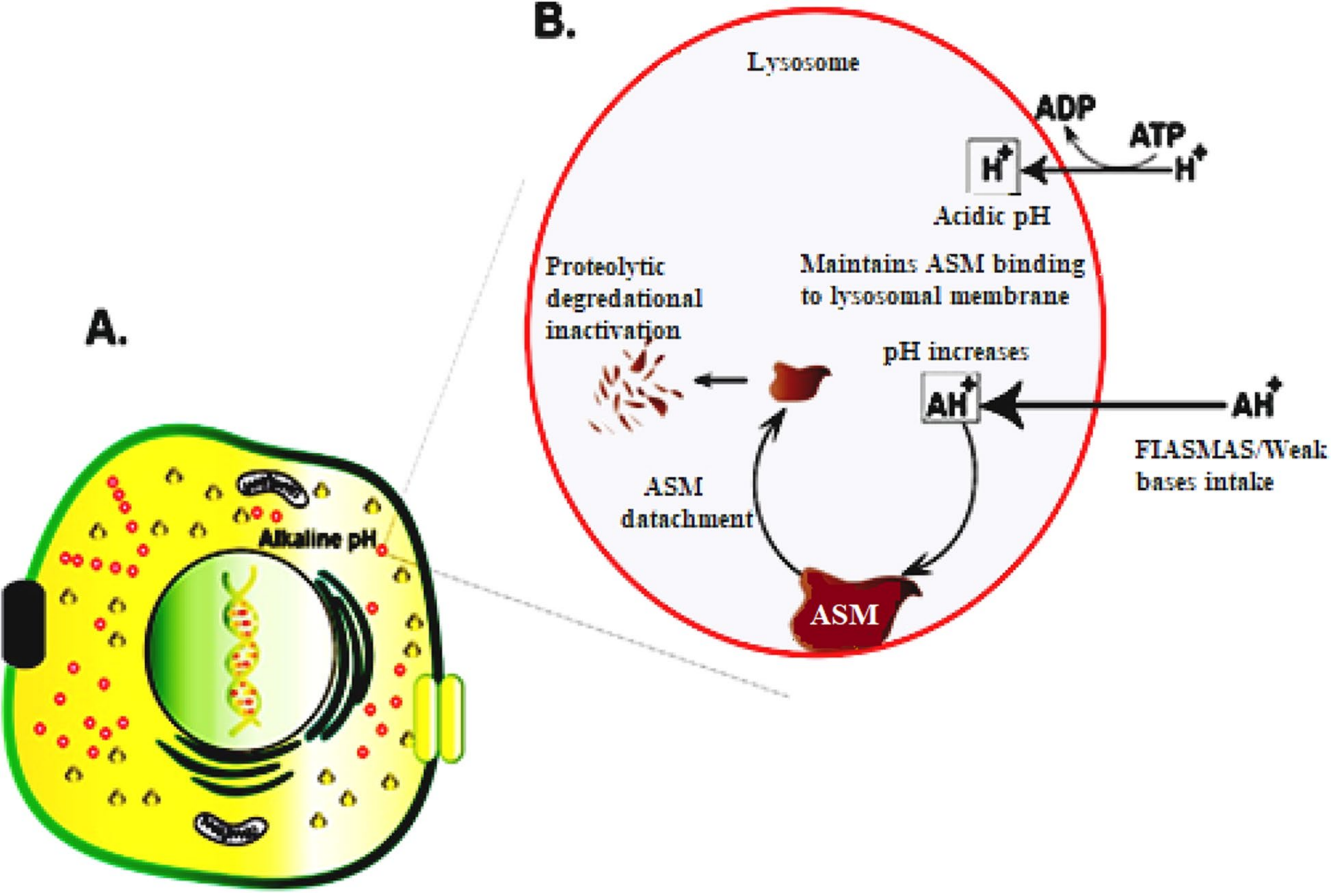
Schematic representation of the mode of action of FIASMAs (functional inhibitors of acid sphingomyelinase). **A** Eukaryotic cell display cellular organelles. **B** A magnified version of lysozyme exhibiting FIASMs indirect inhibition of ASM (acid sphingomyelinase)

Following the detachment, ASMase is cleaved and degraded within the lysosomes by proteolytic degradation [27, 167]. Notably, inhibition of ASMase by certain drugs has long been recognized, but systematic studies describing FIASMA inhibition are fairly new [127] (Figs. 5, 6).

## In Vitro Docking of Potent Antiviral Compounds Based on Sphingolipid Inhibition

Molecular Operating Environment (MOE) software was used to perform docking analyses [168] of acid sphingomyelinase inhibitors to quantify their inhibitory effect on SARS-CoV-2 uptake. Their binding modes with mammalian acid sphingomyelinase’s binding site (PDB code: 5FI9) and their interaction with key amino acids were compared to the candidate drug amiodarone [169]. All structure minimizations were performed until an RMSD gradient of 0.05 kcal∙mol^−1^ Å^−1^ with *MMFF94x* force field, and partial charges were automatically calculated. All intervening water molecules were removed from the structure, and then the target protein was prepared for docking using *Protonate 3D* protocol in MOE with default parameters. The co-crystalized ligand was used to define the binding site for docking simulation. The *Triangle Matcher Placement* method and *London dG* scoring function were employed for docking and scoring. The docking protocol was first validated by self-docking the co-crystallized ligand near the protein's binding site. The ligand-receptor interactions at the protein binding site were studied with the validated docking protocol (RMSD < 2) for the reported inhibitors to predict their binding mode and binding affinity.

**Fig. 7:**
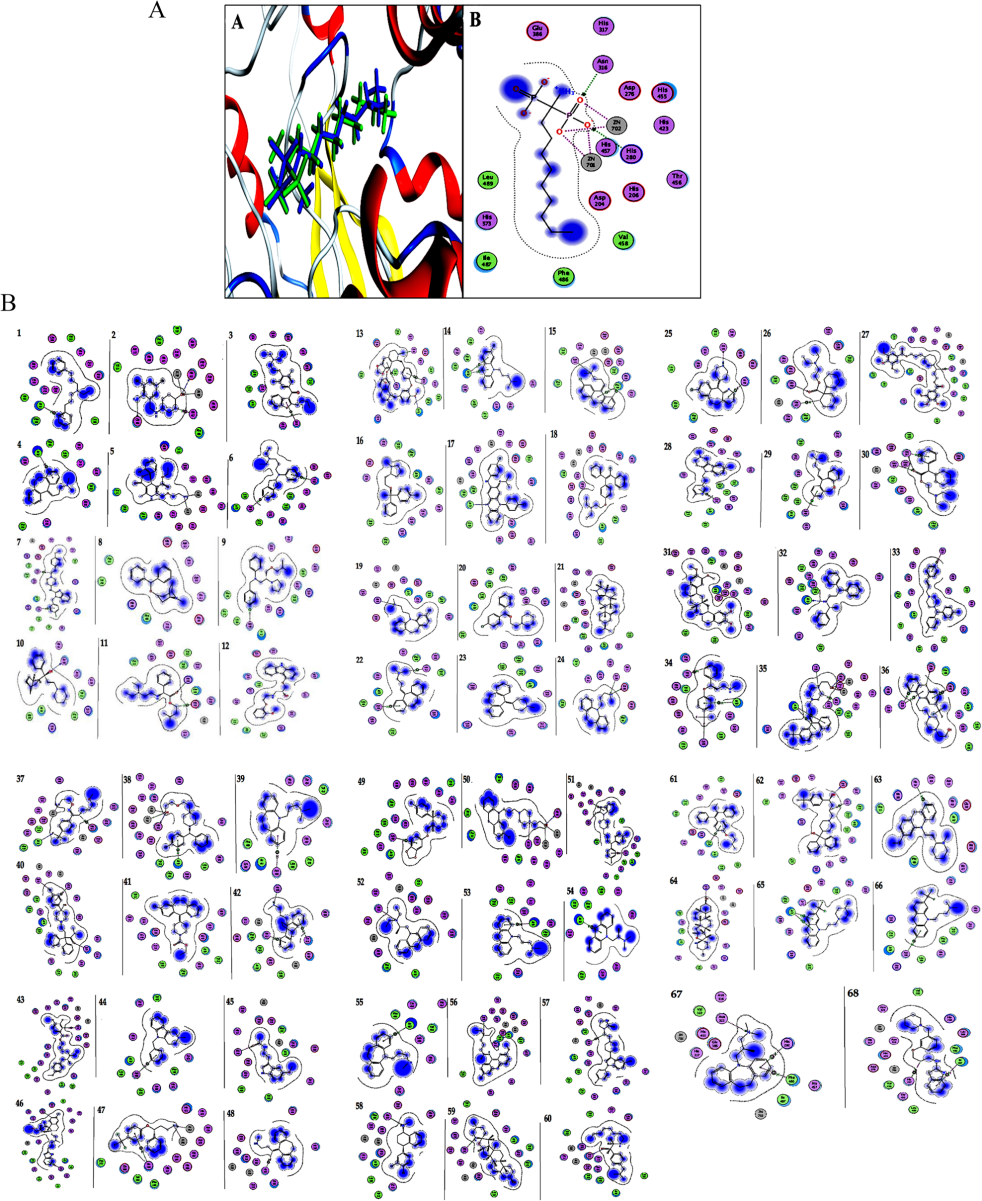
**A** (i) Superimposition of the docking pose (green) and the co-crystallized (blue) of (1-azanyl-1-phosphono-decyl) phosphonic acid (APPA) in the acid sphingomyelinase active site with RMSD of 1.49 Å. (ii) 2D interaction diagram showing APPA docking pose interactions with the hot spots in the enzyme active site. **B**. 2D diagrams of the candidate drug (amiodarone) and the reported inhibitors. 1. Alverine, 2. Ambroxol, 3. Amiodarone, 4. Amitriptyline, 5. Amlodipine, 6. Aprindine, 7. Astemizole,8.Benztropine, 9. Bepridil, 10. Biperidene, 11. Camylofine, 12. Carvedilol, 13. Cepharanthine, 14. Chlorpromazine, 15. Chlorprothixene, 16. Clemastine, 17. Clofazimine, 18. Clomiphene, 19. Clomipramine, 20. Cloperastine, 21. Conessine, 22. Cyclobenzaprine, 23. Cyproheptadine, 24. Desipramine, 25. Desloratadine, 26. Dicycloverine, 27. Dilazep, 28. Dimebon, 29. Doxepine, 30. Drofenine, 31. Emetine, 32. Fendeline, 33. Flunarizine, 34. Fluoxetine, 35. Flupenthixol, 36. Fluphenazine, 37. Fluvoxamine, 38. Hydroxyzin, 39. Imipramine, 40. Loperamide, 41. Loratadine, 42. Maproteline, 43. Mebeverine, 44. Mebhydrolin, 45. Melatonin, 46. Mibefradil, 47. Norfluoxetine, 48. Nortriptyline, 49. Paroxetine, 50. Perphenazine, 51. Pimozide, 52. Profenamine, 53. Promazine, 54. Promethazine, 55. Protriptyline, 56. Quinacrine, 57.Sertindole, 58. Sertraline, 59. Solasodine, 60. Suloctidil, 61. Tamoxifene, 62. Terfenadine, 63. Thioridazine, 64. Tomatidine, 65. Trifluoperazine, 66. Triflupromazine, 67. Trimipramine, 68. Zolantidine showing their interaction with the key amino acids in the acid sphingomyelinase

Validation and endorsement of the docking protocol were achieved by self-docking of the co-crystallized (1-azanyl-1-phosphono-decyl) phosphonic acid (APPA) within the acid sphingomyelinase active site with an energy score (S) of –28.75 kcal/mol and RMSD of 1.49 Å, and with reproducing all interactions of APPA with the binding site of the enzyme (Fig. 7A). Reported inhibitors interacted with the key amino acids in the acid sphingomyelinase active site, indicating their inhibition activities as confirmed by their docking scores (S) and binding modes compared to that of the candidate drug amiodarone (Figures. 7B and Table 3).

**Table 3:** Docking energy scores (S) and hot spots involved in binding for APPA (the co-crystalized compound), amiodaron (the drug candidate), and the reported compounds in the acid sphingomyelinase active site

Compound	Docking score (S) (kcal/mol)	Hot spots involved in binding
APPA (Co-crystalized ligand)	− 28.75	Asn316, His280, and Zn(II) ions
Amiodarone (Drug candidate)	− 10.43	Tyr572
Alverine	− 8.22	Ile 487
Astemizole	− 10.81	Asn488 and His457
Aprindine	− 8.41	Asn488 and Thr456
Amlodipine	− 9.14	Zn(II) ions
Ambroxol	− 8.54	His455, His457, Glu386, and Zn(II) ions
Amitriptyline	− 7.94	Ile 487
Benztropine	− 8.18	––––-
Bepridil	− 10.47	Asn488
Biperidene	− 7.96	Thr456
Camylofine	− 8.55	His280 and His455
Carvedilol	− 11.28	––––-
Cepharanthine	− 11.06	Asn316, His280, Ile487, and His457
Clofazimine	− 10.37	Ile 487
Clemastine	− 8.40	––––-
Cloperastine	− 8.04	––––-
Chlorprothixene	− 7.86	Asn323, His280, and Phe486
Chlorpromazine	− 8.29	Ile 487
Clomiphene	− 8.95	––––-
Clomipramine	− 8.31	His280
Conessine	− 8.33	––––-
Cyclobenzaprine	− 8.03	Asn488 and His457
Cyproheptadine	− 8.45	––––-
Desipramine	− 8.38	Asn316 and Glu386
Desloratadine	− 8.77	Asn323 and His280
Dicycloverine	− 7.53	His280 and His457
Dilazep	− 12.58	His457
Dimebon	− 9.41	Asn488
Doxepine	− 8.36	Asn488
Drofenine	− 8.08	Asn316 and His317
Emetine	− 11.65	––––-
Fendeline	− 9.06	Ile487
Flupenthixol	− 10.39	His455, His280, His457, Ile487, and Zn(II) ions
Fluoxetine	− 10.09	His457, Ile487, and Lys103
Fluvoxamine	− 9.37	His455, Ile487, and Zn(II) ion
Fluphenazine	− 9.59	His455, His317, and Glu386
Flunarizine	− 9.20	His317
Hydroxyzine	− 10.96	His455, Ile487, and Zn(II) ions
Imipramine	− 7.76	Asn488
Loperamide	− 10.13	Asn316, His280, and Lys103
Loratadine	− 8.47	––––-
Maproteline	− 7.96	His280, Thr456, and His457
Melatonine	− 9.23	His280
Mebhydroline	− 8.02	Asn488
Mebeverine	− 11.14	His457
Mibefradil	− 10.09	Asn488 and Glu386
Norfluoxetine	− 10.34	His280 and Zn(II) ions
Nortriptyline	− 7.49	His457
Paroxetine	− 10.23	Asn488
Perphenazine	− 9.78	His455, His317, and Zn(II) ions
Pimozide	− 11.29	His280, His317, and Asn488
Profenamine	− 7.72	His317
Promethazine	− 7.62	Ile487
Promazine	− 8.09	Ile487
Protriptyline	− 8.48	Ile487
Quinacrine	− 10.19	His280
Sertindole	− 10.55	His457
Solasodine	− 8.64	His317
Sertraline	− 7.77	His317
Suloctidil	− 8.64	His455
Tamoxifene	− 8.56	Phe486
Thioridazine	− 8.00	His317
Tomatidine	− 8.85	His280
Terfenadine	− 10.39	His455 and His457
Trifluoperazine	− 10.22	Ile487
Triflupromazine	− 8.72	Asn488
Trimipramine	− 7.79	His280, Asn323, and Phe486
Zolantidine	− 9.13	His457 and Ile487

The docking simulation studies revealed that dilazep (S = − 12.58 kcal/mol), emetine (S = − 11.65 kcal/mol), pimozide (S = − 11.29 kcal/mol), carvedilol (S = − 11.28 kcal/mol), mebeverine (S = − 11.14 kcal/mol), cepharanthine (S = − 11.06 kcal/mol), hydroxyzine (S = − 10.96 kcal/mol), astemizole (S = − 10.81 kcal/mol), sertindole (S = − 10.55 kcal/mol), and bepridil (S = − 10.47 kcal/mol) had higher inhibition activity than the candidate drug amiodarone (S = − 10.43 kcal/mol) towards the acid sphingomyelinase. In addition, dilazep (S = − 12.58 kcal/mol) was the most effective inhibitor. Additionally, we provided a comprehensive Table 4 for publicly available inhibitors of ASMase in vitro and in vivo in previous studies to give insight into experimental data regarding ASMase inhibitors.

**Table 4:** In vitro and in vivo studies of FIASMAs

FIASMAs	In silico study	In vitro study	In vivo study	Refences
Alverine	-	Show functional inhibition of ASMase with residual ASM activity of 21.7	-	[27]
Astemizole	Astemizole formed one hydrogen bond with ACE2 while three hydrogen bonds with H1R. Nitrogen on the hexahydropyridine ring of astemizole forms hydrogen bonds with ARG393 of ACE2 with distances of 2.14 Å. Asmidazole forms hydrogen bonds with LYS1016, ANS1055, and ASN1053 of H1R with distances of 1.92 Å, 2.39 Å, and 1.91 Å, respectively	The results showed that astemizole can bind to the ACE2 receptor and inhibit the invasion of SARS-COV-2 Spike pseudoviruses	–	[170]
Aprindine	-	Show functional inhibition of ASMase with residual ASM activity of 27.5	–	[27]
Amlodipine	Amlodipine showed binding affinity to S glycoprotein and 3-chymotrypsin-like protease was − 5.5, − 6.0, and − 5.2, respectively	Amlodipine Besylate showed antiviral activity against OC43 cells through binding and acting as a carbonic anhydrase inhibitor, calcium channel inhibitor, and PDE inhibitor	Chronic treatment with amlodipine could be significantly associated with low mortality of COVID-19 in patients	[171] [172]
Ambroxol	––-	––––	The system of sphingomyelinase/ceramide is very significant in transmitting SARS-CoV-2. They used Ambroxol, which has trans-4-[(2,4-dibromanilin-6-yl)-methyamino]-cyclohexanol structure as an inhibitor of ASMase. The Ambroxol is applied by inhalation, suggesting that the drug might inhibit the acid sphingomyelinase and, thereby, infection with SARS-CoV-2. They used vesicular stomatitis virus pseudoviral particles presenting SARS-CoV-2 spike protein on their surface (pp-VSV-SARS-CoV-2 spike), a *bona fide* system for mimicking SARS-CoV-2 entry into cells. They found that entry of pp-VSV-SARS-CoV-2 spike required activation of acid sphingomyelinase and release of ceramide, all of which were prevented by pretreatment with ambroxol. They also obtained nasal epithelial cells from human volunteers before and after inhalation of ambroxol. Inhalation of ambroxol reduced acid sphingomyelinase activity in nasal epithelial cells and prevented pp-VSV-SARS-CoV-2 spike-induced acid sphingomyelinase activation, ceramide release, and entry of pp-VSV-SARS-CoV-2 spike *ex vivo* [123]	[123]
Amiodarone	–	Amiodarone reduced SARS-CoV-2 and IAV titres ≥ 90% without any cytotoxic effects. It also inhibited SARS2 replication, reducing supernatant viral RNA load with a promising activity level	Amiodarone administration in an early disease phase might block SARS-CoV-2 replication	[156]-[173]
Amitriptyline	Amitriptyline showed binding to the allosteric site of SARS-CoV-2 Main Protease with − 5.9 kcal/mol	The results showed that the increased ASMase activity and ceramide release were inhibited by pretreatment with Amitriptyline at 0.625, 1.25, 2.5, and 5 μM. Thus, amitriptyline was regarded as an active inhibitor of ASMase	In healthy volunteers, oral administration of amitriptyline blocked infection of freshly isolated nasal epithelial cells with SARS‐CoV‐2	[174–176]
Benztropin	-	Benztropin inhibited ASMase activity by at least 50% at 10 µM	In healthy volunteers, oral administration of amitriptyline blocked infection of freshly isolated nasal epithelial cells with SARS‐CoV‐2	[174, 177]
Bepridil	Amitriptyline showed binding to the allosteric site of SARS-CoV-2 Main Protease with − 5.1 kcal/mol	Bepridil possesses significant anti − SARS-CoV-2 activity in both Vero E6 and A459/ACE2 cells in a dose-dependent manner with low micromolar effective concentration 50% (EC_50_) values	-	[178]
Biperidene	-	Showed inhibitory impact on ASMase	-	[174]
Camylofine	-	Camylofin showed an inhibitory impact with a pKa of 10.02	-	[127]
Carvedilol	-	-	Carvedilol usage was not significantly associated with a reduced likelihood of a positive laboratory test result for SARS-CoV-2 among the 5 subgroups after adjusting for age, sex, race, smoking, and various disease comorbidities	[130]
Cepharanthine	Cepharanthine can block both the NSP12‐NSP7 interface and the NSP12‐NSP8 interface of SARS‐CoV‐2 and the NSP12‐NSP8 interface of SARS‐CoV.2	Cepharanthine showed potential antiviral activities against SARS-CoV-2, with IC_50_ values between 0.1 and 10 μM		[179]
Clofazimine	Clofazimine inhibit 3CL^PRO^	Clofazimine showed IC_50_ value of 0.01 µM	Our data provide evidence that clofazimine may have a role in controlling the current COVID-19 pandemic and, more importantly, in dealing with coronavirus diseases that may emerge	[164, 180, 181]
Clemastine	Clemastin inhibits SARS-CoV-2 replication by non-specific (off-target) effects. Clemastine was docked into the agonist-bound state structure of the receptor (6DK1) with solvation-corrected docking of − 43 kcal/mol	Clemastine inhibited SARS2 replication, reducing supernatant viral RNA load with a promising level of activity with EC50 = 0.95 ± 0.83 µM	-	[136, 137, 182]
Cloperastine	Cloperastine inhibited SARS-CoV-2 replication by non-specific (off-target) effects	-	-	[182]
Chlorprothixene	-	Chlorprothixene inhibits the SARS-CoV replication with EC50s around 10 µM	-	[183]
Chlorpromazine	Chlorpromazine inhibited SARS-CoV-2 replication by non-specific (off-target) effects	Chlorpromazine didn’t inhibit the virus replication	Inhibited viral replication in the lungs but protected against SARS-CoV-2	[184, 185]
Clomiphene	-	Clomiphene showed an inhibitory impact with IC50 of 3.32 µM	-	[142]
Clomipramine	-	Clomipramine showed an IC50 average of 5.63	-	[184]
Conessine	-	Show functional inhibition of ASMase with residual ASM activity of 20.8	-	[27]
Cyclobenzaprine	-	-	-	
Cyproheptadine	-	-	-	
Desipramine	-	Desipramine with concentrations of 5 μM and 35 μM inhibited acid sphingomyelinase activity	-	[186]
Desloratadine	-	Desloratadine, a commonly used antiallergic, well-tolerated with no major side effects, potently reduced the production of SARS-CoV-2 RNA in Vero-E6 cells	Finally, the ex vivo kinetic of the antiviral effect of desloratadine was evaluated on primary Human Nasal Epithelial Cells (HNEC), showing a significant delay of viral RNA production with a maximal reduction reached after 72 h of treatment	[187]
Dicycloverine	-	Dicycloverine, showed antiviral efficacy against SARS-CoV-2, reducing viral infection by at least 50%,	-	[188]
Dilazep	-	-	-	
Dimebon	-	Inhibited ASMase with residual activity 44.1%	-	[27]
Doxepine	-	Doxepin could inhibit SARS-CoV-2 spike pseudovirus from entering the ACE2-expressing cell, reducing the infection rate to 25.82%	-	[189]
Drofenine	-	Drofenine showed an inhibitory impact through pKa alteration of 9.21	-	[127]
Emetine	Emetine (P5) showed binding energy to RNA-dependent RNA polymerase (RdRp) enzyme with − 7.81 kcal/mol	Antiviral effect of emetine against SARS-CoV-2 virus in Vero E6 cells with the estimated 50% effective concentration at 0.46 μM	-	[148, 190],
Fendeline	-	-	-	
Flupenthixol	Flupenthixol showed docking PLANTS score with RdRp and MPro with − 91.70 and − 91.82, respectively	Antiviral tests using native SARS-CoV-2 virus in Vero E6 cells confirmed that flupenthixol significantly inhibited SARS2 replication, reducing supernatant viral RNA load with a promising activity level	Flupenthixol inhibited viral entry in our lung organoid model	[136, 191]
Fluoxetine	Fluoxetine demonstrates non-serotonergic, anti-inflammatory effects. Our results show a critical role for IL6 signal transduction protein (IL6ST) and NF-kappaB Subunit 1 (NFKB1) in fluoxetine’s ability to act as a potential therapy for hyperinflammatory states such as asthma, sepsis, and COVID-19	Fluoxetine with concentrations between 5 μM and 35 μM inhibited acid sphingomyelinase activity	In this multicenter retrospective observational study involving a large sample of patients hospitalized for COVID-19, we found that antidepressant use, at a mean dosage of 21.6 (SD = 14.1) fluoxetine-equivalent milligrams, was significantly and substantially associated with reduced risk of intubation or death, independently of patient characteristics, clinical and biological markers of disease severity, and other psychotropic medications	[144, 186]
Fluvoxamine		Fluvoxamine reduced the viral infection, as measured by luciferase reporter activity	Treatment of COVID-19 patients with fluvoxamine for 2 weeks also effectively decreased the development of clinical deterioration	[143, 152]
Fluphenazine	Fluphenazine revealed the best binding pattern and the highest docking score against the main protease binding site (–11.75 kcal/mol)	Fluphenazine dihydrochloride showed IC_50_ (Avg) of 6.36 against against SARS-CoV-2	-	[153, 184]
Flupentixol	-	-	-	
Flunarizine	Flunarizine by a spike protein docking screen	Flunarizine showed an impact against SARS-CoV-2, which was confirmed through cytopathic effect (CPE) assay in Vero E6 cells with EC50 (uM) of 10.0	-	[185, 192]
Hydroxyzin	The drugs that passed all applied lysosomotropism criteria are azithromycin, promethazine, cyclizine, chloroquine, clemastine, hydroxyzine, rifabutin and vicriviroc, and drugs that do not have data for one of the criteria but passed all the others are chlorcyclizine, homochlorcyclizine and quinacrine	The diphenhydramine, hydroxyzine, and azelastine to exhibit direct antiviral activity against SARS-CoV-2 in vitro	Usage of hydroxyzine was associated with reduced incidence of SARS-CoV-2 positivity in subjects greater than age 61	[154, 193]
Imipramine	Inhibitor candidate for SARS-CoV-2 Main Protease	Concentrations between 5 μM and 35 μM inhibited acid sphingomyelinase activity	-	[176, 186]
Loperamide	-	Loperamide hydrochloride showed antiviral effect against In vitro live virus	-	[179]
Loratadine		In vitro, severe acute respiratory syndrome coronavirus-2 (SARS-CoV-2) spike pseudotyped viral infection experiments indicated that histamine H1 antagonists loratadine (LOR) and desloratadine (DES) could prevent the entry of the pseudotyped virus into ACE2-overexpressing HEK293T cells and showed that DES was more effective	Prior usage of loratadine was associated with a reduced incidence of positive SARS-CoV-2 test results in individuals 61 years and above in a statistically significant manner	[154, 158]
Maproteline	-	-	-	
Melatonine	The establish that a combinatorial drug treatment using melatonin and toremifene will provide an effective therapeutic strategy to mitigate the severity of COVID-19 In summary, combining mercaptopurine and melatonin may offer a potential combination therapy for 2019-nCoV/SARS-CoV-2 by synergistically targeting papain-like protease, ACE2, c-Jun signalling, and anti-inflammatory pathways		The risk was reduced in those who had pneumococcal polysaccharide or influenza vaccine or were on melatonin, paroxetine, or carvedilol	[160, 194, 195]
Mebhydroline	-	Mebhydroline causes in vitro inhibition of acid sphingomyelinase	-	[174]
Mebeverine	-	-	-	
Mibefradile	-	Mibefradile causes in vitro inhibition of acid sphingomyelinase	-	[174]
Norfluoxetine	-	-	-	
Nortriptyline	The potential to reverse transcriptomic signature upon SARS-CoV-2 through acting as an antagonist for Adrenergic uptake inhibitor	-	-	[196]
Paroxetine	-	-	Most potentially impactful is the reduced risk of testing positive in patients who were on melatonin, carvedilol, and paroxetine, which are drugs identified in drug-repurposing studies to have a potential benefit against COVID-19	[160]
Perphenazine	-	-	-	
Pimozide	Pimozide, tested by computational docking analysis and in vitro assays, has been suggested to inhibit the main protease of SARS-CoV-2 (MPro)	Pimozide, ebastine, and bepridil were the three most potent FDA/EMA-approved medicines, with IC50 values of 42 ± 2, 57 ± 12, and 72 ± 12 µM, respectively Pimozide inhibited the infection by pseudotyped viruses with minimal effects on cell viability	-	[143, 178]
Pimethexene	-	-	-	
Profenamine	Profenamine showed binding affinity to ASMase of − 8.7 kcal/mol	-	-	[197]
Promethazine	-Promethazine showed effectiveness against either SARS-CoV, SARS-CoV-2 or MERS viruses or two or all of them, supporting the potential value of this antiviral strategy -Promethazine is a candidate for targeting COVID-19 Related Genes	Promethazine hydrochloride showed IC50 (avg) of 9.21 μM	-	[184, 193, 198]
Promazine	-	Promazine was identified as a high-confidence inhibitor of SARS-CoV-2 replication	-	[199]
Protriptyline	-	-	-	
Quinacrine	The remaining top candidate drugs identified by our analysis include kinase inhibitors erlotinib, alvocidib, dasatinib, antimalarial quinacrine, and phenothiazine thioridazine, a more commonly used antipsychotic. These drugs also have antiviral properties and are yet to be explored for the treatment of COVID-19	-	-	[200]
Sertindole	-	Sertindole showed in vitro inhibition of acid sphingomyelinase	-	[174]
Solasodine	Solasodine showed a binding affinity of − 8.7 against ASMase	-	-	
Sertraline	-	Mechanistically, sertraline HCl was found to block SARS-CoV-2 S protein-mediated cell fusion	-	[164]
Suloctidil	-	-	-	
Tamoxifene	Overall, we recommend that tamoxifen may protect against cytokine storms, alleviate ARDS in COVID-19 patients, and reduce the incidence of critical illness and mortality	Tamoxifen citrateshowed IC50 (avg) of 34.12 μM	-	[184, 198]
Thioridazine	Thioridazine and its identified photoproducts (mesoridazine and sulforidazine) have high biological activity on the virus Mpro. This shows that thioridazine and its two photoproducts might represent new potent medicines to be used for treatment in this outbreak -	Thioridazine has anti-SARS-CoV-2 activity in vitro	-	[164, 201]
Tomatidine	Profenamine showed binding affinity to ASMase of − 8.7 kcal/mol	-	-	
Terfenadine	-	Terfenadine can reverse the transcriptional landscape induced by SARS-CoV-2 infection when tested on Vero-E6 cells infected with SARS-CoV-2 and on human pluripotent stem-cell-derived pancreatic endocrine organoid cultures	-	[202]
Trifluoperazine	Trifluoperazine was predicted to bind to Mpro and RdRp (PLANTS scores < − 80.00), thus corroborating putative multimodal actions	Trifluoperazine 2HCl showed antiviral activity against SARS-CoV-2 with CC50 and IC50(μM) of 29.29 and 11.75, respectively	-	[164, 191]
Triflupromazine	-	The triflupromazine demonstrated antiviral activity in a screen against MERS-CoV replication in Huh-7 cells	-	[24]
Trimipramine	Amitriptyline showed binding to the allosteric site of SARS-CoV-2 Main Protease with − 5.5 kcal/mol	-	-	[176]
Zolantidine	-	-	-	

(-) = Not found

## Conclusion and limitations

Nevertheless, dilazep showed the most promising in silico results against ASMase with (S = − 12.58 kcal/mol); we couldn’t find a correlation with experimental data; however, our pre-elementary docking can be validated through in vitro and in vivo future experimental data. Interestingly, emetine had (S = − 11.65 kcal/mol), consistent with its in vitro capacity against SARS-CoV-2 virus in Vero E6 cells with the estimated 50% effective concentration at 0.46 μM [148]. Pimozide pointed out (S = − 11.29 kcal/mol) can be correlated with its IC_50_ potency of 42 ± 2 µM and its potent inhibitory infection by pseudotyped viruses with minimal effects on cell viability [143, 178]. While carvedilol had (S = − 11.28 kcal/mol), a previous cohort study didn’t confirm its role as a significant player against SARS-CoV-2 [130]. Mebeverine showed (S = − 11.14 kcal/mol); however, to our knowledge, the inhibitor hasn’t been tested experimentally. Furthermore, cepharanthine, which pointed out (S = − 11.06 kcal/mol), had potential antiviral activities against SARS-CoV-2 [179]. Hydroxyzine (S = − 10.96 kcal/mol) had previously shown a significant impact against SARS-CoV-2 in vitro and in vivo approaches [154, 193]. Astemizole had (S = − 10.81 kcal/mol) given by its ability to bind to the ACE2 receptor and inhibit the invasion of SARS-COV-2 Spike pseudoviruses [170]. Sertindole had (S = − 10.55 kcal/mol) results, which is in agreement with its showed in vitro inhibition of acid sphingomyelinase [174]. Bepridil (S = − 10.47 kcal/mol) was found to be a significant inhibitor against SARS-CoV-2 activity in both Vero E6 and A459/ACE2 cells in a dose-dependent manner with low micromolar effective concentration, 50% (EC_50_) values [178].

### Limitations

Our work can be considered pre-elementary screening for ASMase inhibitors, leading to several candidates that should be tested in vitro and in vivo. Also, FIASMAs binding to the active site of ASMase wasn’t significantly robust as compared to crystallographic ligand (S = –28.75 kcal/mol), which can be attributed to the indirect work of FIASMAs through lysosomal accumulation and raising intra-lysosomal pH causing reduced the electrostatic interactions between the lysosomal membrane and the ASMase, resulting in ASMase detachment. Also, our in silico framework depended only on MOE software, which didn’t reveal the standard deviations of binding energies, so we recommend using additional software to validate the results further.

## Data Availability

The data supporting this study's findings are available from the corresponding author upon reasonable request.
